# The use of prostate specific antigen density to predict clinically significant prostate cancer

**DOI:** 10.1038/s41598-020-76786-9

**Published:** 2020-11-17

**Authors:** Igor Yusim, Muhammad Krenawi, Elad Mazor, Victor Novack, Nicola J. Mabjeesh

**Affiliations:** 1 Department of Urology, Soroka University Medical Center, Faculty of Health Sciences, Ben-Gurion University of the Negev, Be’er-Sheva, Israel; 2 Soroka Clinical Research Center, Soroka University Medical Center, Faculty of Health Sciences, Ben-Gurion University of the Negev, Be’er-Sheva, Israel; 3 Department of Urology, Soroka University Medical Center, POB 151, 84101 Be’er-Sheva, Israel

**Keywords:** Prostate, Urology, Oncology, Urological cancer, Prostate cancer

## Abstract

The purpose of this study was to assess the predictive value of prostate specific antigen density (PSAD) for detection of clinically significant prostate cancer in men undergoing systematic transrectal ultrasound (TRUS)-guided prostate biopsy. We retrospectively analyzed data of men who underwent TRUS-guided prostate biopsy because of elevated PSA (≤ 20 ng/ml) or abnormal digital rectal examination. Receiver operating characteristic curve analysis to compare PSA and PSAD performance and chi-square automatic interaction detector methodologies were used to identify predictors of clinically significant cancer (Gleason score ≥ 7 or international society of urological pathology grade group ≥ 2). Nine-hundred and ninety-two consecutive men with a median age of 66 years (IQR 61–71) were included in the study. Median PSAD was 0.10 ng/ml^2^ (IQR 0.10–0.22). Prostate adenocarcinoma was diagnosed in 338 men (34%). Clinically significant prostate adenocarcinoma was diagnosed in 167 patients (50% of all cancers and 17% of the whole cohort). The AUC to predict clinically significant prostate cancer was 0.64 for PSA and 0.78 for PSAD (P < 0.001). The highest Youden's index for PSAD was at 0.20 ng/ml^2^ with 70% sensitivity and 79% specificity for the diagnosis of clinically significant cancer. Men with PSAD < 0.09 ng/ml^2^ had only 4% chance of having clinically significant disease. The detection rate of clinically significant prostate cancer in patients with PSAD between 0.09 and 0.19 ng/ml^2^ was significantly higher when prostate volume was less than 33 ml. In conclusion, PSAD was a better predictor than PSA alone of clinically significant prostate cancer in patients undergoing TRUS-guided biopsy. Patients with PSAD below 0.09 ng/ml^2^ were unlikely to harbor clinically significant prostate cancer. Combining PSAD in the gray zone (0.09–0.19) with prostate volume below 33 ml adds diagnostic value of clinically significant prostate cancer.

## Introduction

Prostate cancer is recognized as one of the (leading) diseases in the male population ^1^. Early detection of clinically significant prostate cancer is very important in order to achieve high rates of cure and local disease control. Historically, digital rectal examination (DRE) was the only tool used in the diagnosis of prostate cancer and in particular locally advanced disease. The accuracy of DRE in detecting prostate cancer is very low and it is rather used for clinical staging ^2^. After prostate-specific antigen (PSA) emerged, its use was immediately coupled with DRE to improve the detection of prostate cancer unfortunately this resulted in unnecessary earlier diagnosis and over treatment of patients with very low and low risk disease ^3^. Therefore, several measures have been proposed to improve the specificity of the PSA test, including, PSA velocity, free-to-total PSA ratio and PSA density (PSAD). Since PSAD introduction in the early 1990s by Benson et al., ^4^ it has been demonstrated to be a better predictor of prostate cancer than PSA but its application was not consistent in daily practice over the years ^5,6^.

At the same time in the modern era of the emerging role of prebiopsy diagnostic MRI to evaluate potential prostate pathology, noninvasive methods that reduce unnecessary biopsies are warranted. The need for such methods is higher in populations where MRI availability is limited. As the PSA blood test nowadays is part of our daily practice and the calculation of prostate volume is relatively simple, the application of PSAD would be possible in order to identify high risk prostate cancer. A similar trend was recently shown when the Stockholm-3 model, combining clinical, genomic variables and blood biomarkers, was thoroughly analyzed it found PSAD to improve the total performance of the model ^7^. A number of recent reports also reexamined the added value of PSAD to detect clinically significant cancer and further refining a PSAD cutoff for this purpose ^8−12^.

In this current study, we evaluated the value of PSAD in predicting clinically significant prostate cancer in our population.

## Patients and methods

### Patients

Soroka University Medical Center ethics committee approved the study and waived informed consent requirements and all methods were performed in accordance with the relevant guidelines and regulations. We retrospectively collected data on 1,226 consecutive men who underwent TRUS-guided prostate biopsy at our center between January 2014 and December 2018. Men with PSA levels of > 4 ng/ml, or with abnormal DRE findings, were referred for TRUS-guided prostate biopsy. Ideally, prostate biopsies were performed with 12 cores taken from both lobes, 6 from each side, under TRUS guidance; two specimens were obtained from the base of the prostate gland of each side, two from middle of the gland, and two from the apex. Occasionally, more cores were taken when suspicious lesion was noticed on ultrasonography. All biopsies were performed only by experienced senior urologists. Since repeat biopsies for the same patients were included in the database, we selected the first available biopsy for each patient performed at our center and we excluded all repeated biopsies. Biopsy specimens were reviewed by experienced pathologists. Our cohort included 992 men with the restriction of PSA ≤ 20 ng/ml measured prior to biopsy. Estimated prostate volume was measured by TRUS. The PSAD (ng/ml^2^) was calculated as PSA (ng/mL) divided by the prostate volume (ml).

### Statistical analysis

Descriptive statistics of the study sample were used to summarize relevant participant characteristics. The Student’s t-test was used for comparison of the continuous variables. Chi-square and Fisher’s exact tests were used for comparison of the proportions. Receiver operating characteristic (ROC) curves were plotted as sensitivity vs. 1-specificity for PSA and PSAD for each outcome. Clinically significant prostate cancer is defined as Gleason score ≥ 7 or international society of urological pathology (ISUP) grade group 2 or greater. We calculated the PSA and PSAD AUC predicting clinically significant disease and overall prostate cancer vs. no cancer ^13^. We have used Youden's index (sensitivity + specificity-1) for identification of the optimum cut-off point for PSAD to be used as a predictor of the significant finding. Classification and regression tree and χ2 automatic interaction detection (CHAID) methods were used to divide the predictors into categories on the basis of the clinically significant cancer detection status ^14^. All tests were two-tailed and statistical significance was defined as a P < 0.05. Analyses were performed using SPSS Statistics for Windows, version 24 (IBM Corp., Armonk, N.Y., USA).

## Results

Nine-hundred ninety-two consecutive men with a median age of 66 years (IQR 61–71) who had TRUS-guided prostate biopsy were included in the study. Median PSA was 6.2 ng/ml (IQR 5–8.3) and median prostate volume was 43 ml (IQR 31–59). Median PSAD was 0.10 ng/ml^2^ (IQR 0.10–0.22). Prostate adenocarcinoma was diagnosed in 338 men (34%).

Table 1 summarizes the clinical and histopathological features of the men with cancer vs. men without cancer. Men with prostate cancer were slightly older (median age of 68 vs. 66) with significantly higher mean PSA and PSAD values but lower mean prostate volume. Of the men with cancer about 50% had clinically significant prostate cancer, ISUP grade group ≥ 2 (Gleason score ≥ 7).

**Table 1:** Clinical and pathological characteristics of study patients.

Characteristic	Cancer	No cancer	P-value
No. (%)	338 (34)	654 (66)	
**Age (years)**			
Mean ± SD	67.9 ± 6.9	65.4 ± 6.9	< 0.001
Range	45–84	47–89	
Median (IQR)	68 (63–73)	66 (61–70)	
**Suspicious DRE (%)**	28 (8.3)	26 (4)	0.007
**PSA (ng/ml)**			
Mean ± SD	8.02 ± 4.06	6.60 ± 2.83	< 0.001
Range	1.2–20	0.4–19	
Median (IQR)	6.8 (5.2–9.5)	5.9 (4.9–7.8)	
**Prostate volume (ml)**			
Mean ± SD	37.90 ± 22.6	53.46 ± 25.30	< 0.001
Range	10–198	10–190	
Median (IQR)	32.5 (23–46)	49 (36–65)	
**PSAD (ng/ml**^**2**^**)**			
Mean ± SD	0.27 ± 0.2	0.14 ± 0.1	< 0.001
Range	0.04–1.33	0.006–1.50	
Median (IQR)	0.21 (0.14–0.34)	0.12 (0.09–0.17)	
**Prostate cancer histology**			
ISUP grade group		(%)	
1		49.8	
2		21.6	
3		6.6	
4		15.4	
5		6.6	
Clinical stage		(%)	
T1c		69	
T2a		24	
T2b		2	
T2c		4	
T3a		1	

PSA, prostate-specific antigen; PSAD, PSA density; ISUP, international society of urological pathology. SD, Standard deviation; IQR, interquartile range.

**Figure 1:**
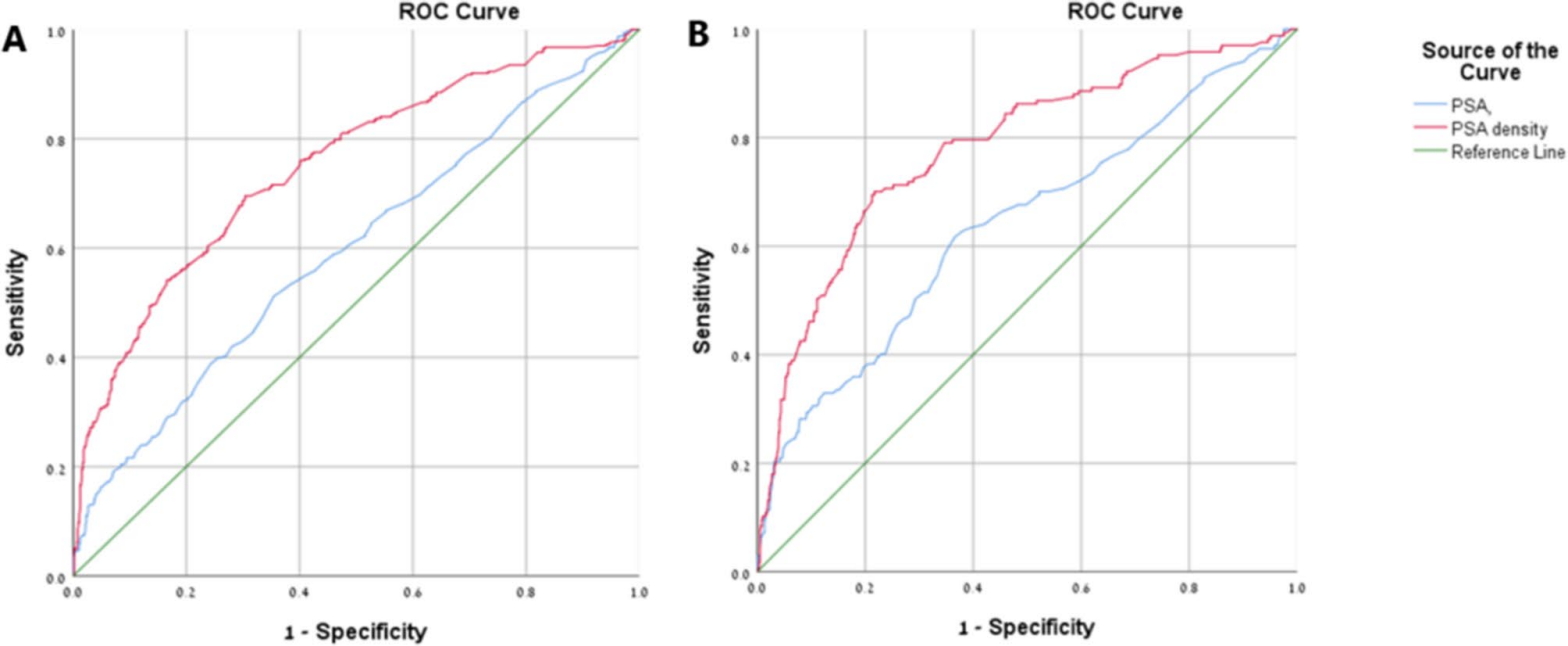
ROC for the comparison between PSA and PSAD for the diagnosis of the prostate cancer. ROC curves comparing PSA and PSAD predictive accuracy to detect overall (**A**) and clinically significant (**B**) prostate cancer. P for comparison between the PSA and PSAD is < 0.001 in A and B. ROC, receiver operating characteristic; PSA, prostate-specific antigen; PSAD, PSA density.

The AUC to predict any prostate cancer was 0.60 (95% CI 0.56–0.63, P < 0.001) for PSA and 0.75 (95% CI 0.72–0.78, P < 0.001) for PSAD (Fig. 1A). The highest Youden's index was at PSAD of 0.15. At this point the diagnosis of cancer has 70% sensitivity and 70% specificity. On the other hand, the AUC to predict clinically significant prostate cancer was 0.64 (95% CI 0.59–0.69, P < 0.001) for PSA and 0.78 (95% CI 0.74–0.82) for PSAD (Fig. 1B). The highest Youden's index was at PSAD of 0.20. At this point the diagnosis of the clinically significant cancer has 70% sensitivity and 79% specificity.

We next used the CHAID methodology to create a decision tree and found that patients with PSAD higher than 0.34 have 56.4% chance to be diagnosed with clinically significant cancer while patients with PSAD less than 0.09 have very low probability (4%) of having clinically significant prostate cancer (Table 2). Men with PSAD between 0.19 to 0.34 have 31.5% chance to be diagnosed with clinically significant prostate cancer. The chance of having clinically significant prostate cancer in men with PSAD between 0.09 and 0.19 is relatively low (8.5%). In this subgroup of patients, when PSAD is combined with prostate volume, the detection rate increases to 16.5% specifically in men having prostate volume less or equal to 33 ml while it drops to 6.8% when prostate volume is larger than 33 ml (Table 2).

**Table 2:**
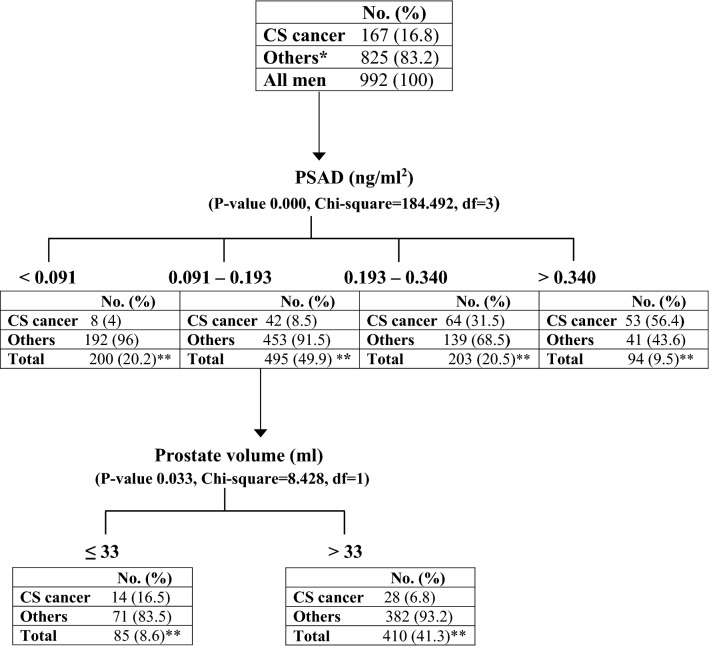
CHAID decision tree for the detection of clinically significant prostate cancer.

*, "Others" consists of patients without cancer and non-clinically significant cancer.

**, percent relates to total number of patients (992).

CHAID, chi-square automatic interaction detector; PSAD, prostate-specific antigen density; CS, clinically-significant.

## Discussion

In the era of post PSA screening there is still great concern about the low reliability of PSA to detect clinically significant prostate cancer. The use of PSAD was first developed by Benson et al. to improve cancer detection rate when PSA ranged between 4 and 10 ng/ml (gray zone) ^15^. Later on, Catalona et al. found high cancer detection performance (95% sensitivity) when they used a PSAD cutoff of 0.078 ng/ml^2^ in men with PSA values in this gray zone ^16^. They found that 40% of cancer cases would have been missed with a cutoff of 0.15 ng/ml^2^ in this population. It should be taken into consideration that PSAD value was originally based on the sextant biopsy template described by Bazinet et al. ^17^, which is not the standard anymore. It is clear that the lower the threshold is set, the more biopsies are done and fewer cancers are missed but the rate of unnecessary biopsies and over detection of non-clinically significant disease are greater.

Recently, Aminsharifi et al. studied the predictive accuracy of PSAD for the detection of clinically significant prostate cancer ^8^. Interestingly, they found that using a PSAD cutoff of 0.08 ng/ml^2^ could have avoided 273 of 2,162 biopsies (13%), missing 48 of 622 non-clinically significant prostate cancers (ISUP grade group 1) (8%) and 10 of 499 clinically significant cancers (2%). Furthermore, Nordström et al. reported that using PSAD cutoffs of 0.1 and 0.15 ng/ml^2^ could lead to missing detection rate of 23% and 51%, respectively, for clinically significant cancers ^9^. But, when they set a cutoff point of 0.07 ng/ml^2^, they were able to avoid about 20% of biopsies while missing only about 7% of clinically significant cancers in patients with PSA 3 ng/ml or greater. It should be kept in mind that these recent studies are based on a higher number of biopsies from the prostate compared to the classic sextant biopsy.

In the current study, the performance of PSA was relatively poor compared with PSAD. Also in our population a cutoff point at PSAD of 0.15 gave the highest sensitivity (70%) and specificity (70%) for the detection of any prostate cancer, while at PSAD of 0.20 gave the highest sensitivity (70%) and specificity (79%) for the detection of clinically significant cancer (Fig. 1).

In addition, we used the CHAID methodology to create a decision tree and found that patients with PSAD higher than 0.19 have 30% to 60% chance to be diagnosed with clinically significant cancer while patients with PSAD less than 0.09 have very low probability (4%) of having clinically significant prostate cancer (Table 2). Interestingly, the chance of having clinically significant prostate cancer in men with PSAD in a "gray zone" between 0.09 and 0.19 is relatively low (8.5%). In this subgroup of patients falling in the PSAD gray zone, the detection rate of clinically significant caner depends on prostate volume. When prostate volume is higher than 33 ml the detection rate is 6.8% whereas it increases more than two-fold to (17%) when prostate volume is smaller than 33 ml (Table 2). The chance of hitting a cancerous lesion is higher in smaller prostates than in larger ones . In our analysis, this was true and significant only in a subgroup of patients with PSAD values between 0.09 and 0.19 indicating that we are not dealing with an artifact. Otherwise, it would have be observed in each value of PSAD.

Our findings are consistent with those recently described by Jue et al. who evaluated the predictive accuracy of PSAD to detect prostate cancer in 1,290 men with various PSA levels ^10^. It should be emphasized that our cohort included patients with PSA levels ≤ 20 ng/ml.

The tendency of PSAD to detect clinically significant prostate cancer has been shown in several clinical scenarios. For instance, the integration of PSAD into active surveillance protocols can be associated with improved enrollment criteria and a reduced rate of upgrading and reclassification down to 17.5% ^11,12^.

It is worth to mention that today there are additional commercially available blood tests to help diagnose clinically significant prostate cancer better than PSA such as 4Kscore and prostate health index (PHI) ^18^ as well as urine-based tests including PCA-3 ^19^ and SelectMDx ^20^.

The limitations of this study that it includes data specific to one population from one area located in southern Israel. Therefore, results may not be extrapolated completely to other populations. Second, this is an observational retrospective study lacking information on the indications for PSA testing which limits our conclusions since no prospective data was collected. Third, the results should be interpreted with caution, since prostate volume was determined only after referral of the patients to TRUS-guided prostate biopsy, but not for the purpose of calculating prostate volume before the decision on the biopsy had been made. Forth, it should be emphasized that only PSA and PSAD were used as predictive variables. Finally, we only included individuals with the first biopsy available without data on repeated biopsies. Therefore, performance and the cutoff point may not be generalizable to candidates for repeat biopsy although PSAD was shown to be a significant predictive factor for positive repeat biopsy ^21^.

In summary, PSAD was a better predictor than PSA alone of biopsy outcomes in patients undergoing transrectal prostate biopsy. Men with PSAD less than 0.09 ng/ml^2^ were unlikely to harbor ISUP grade group 2 or greater disease when PSA was ≤ 20 ng/ml. In patients having PSAD values between 0.09 and 0.19, adjunction of prostate volume increases the performance of PSAD for the detection of clinically significant disease. We propose testing PSAD prior to decision on prostate biopsy since it is a simple inexpensive and available tool that can be used to reduce over detection and morbidity of unnecessary biopsies.

## Ethics approval

Soroka University Medical Center ethics committee approved the study and waived informed consent requirements. We confirm that all methods were performed in accordance with the relevant guidelines and regulations.
